# Emotional Dysregulation Mechanisms in Psychosomatic Chronic Diseases Revealed by the Instability Coefficient

**DOI:** 10.3390/brainsci10100673

**Published:** 2020-09-25

**Authors:** Cristina Ciuluvica (Neagu), Paolo Amerio, Ioan Valeriu Grossu

**Affiliations:** 1 Department of Medicine and Aging Sciences, University G. D’Annunzio, Via dei Vestini, 66013 Chieti, Italy; p.amerio@unich.it; 2 Faculty of Physics, University of Bucharest, P.O. Box MG 11, 077125 Bucharest-Magurele, Romania; ioan.grossu@brahms.fizica.unibuc.ro

**Keywords:** Chaos Theory, emotion dysregulation, psychosomatic disease, cancer, negative affect

## Abstract

In the present work, we analyzed some emotional mechanisms (emotion dysregulation—ED, negative affect—NA, and emotional vulnerability) involved in chronic diseases by means of an interdisciplinary approach. We started from the conceptualization of emotions as a complex dynamic system that can be investigated and understood within a framework inspired by Chaos Theory. An “instability coefficient” Δ was computed to analyze ED mechanisms, NA, and emotional vulnerability in different disease groups (blood cancer, breast cancer, hypertension) as well as in healthy persons. This coefficient, recently defined by our group, computes the Euclidian distance between the pairs of vectors whose components are similar or reverted items of a test measuring ED. The emotional and somatic systems were considered as two complex dynamical systems in interaction. Due to this interaction, and as a result of the laws of complexity, a small perturbation in an inner state of the emotional system could generate an important reaction in the somatic system in time. The emotional vulnerability reflected by high values of Δ was associated with the chronic disease condition. The differences between illness groups and healthy persons, as well as between the three disease groups in Δ values, were analyzed. The results showed that there were significant differences between the chronic disease groups in Δ values. The most highly significant differences in Δ values were reported between the breast cancer group and the healthy group on one hand and between the breast cancer group and the blood cancer group on the other hand. The less significant differences in Δ values were noticed between the hypertension group and the control group. Δ was significant in predicting ED and NA. Compared to the classical approaches, the original contribution of our research is that these results encourage us to propose this interdisciplinary method of assessment as a challenging, valid tool of investigation and understanding of complex phenomena that occur in the emotional and somatic system.

## 1. Introduction

In the last decade, the progress that the field of physics has achieved in understanding complex systems’ dynamics has found applications in other domains (such as biology, psychology, medicine, etc.). In this context, the theoretical framework of dynamic systems was used to investigate and predict the self-organization mechanisms in complex systems that constantly change, reorganize, and progress over time [1,2,3,4]. The self-organization is a spontaneous evolution process of complex systems that emerges only from the interactions between the basic components of the system, without any specific outside intervention. The system evolves from an apparent state of chaos to a qualitatively stable structure. This evolution occurs by continuous feedback loops among multiple systems that do not require cognizant awareness of the relationships between their different components [5,6,7,8,9].

In the present work, the emotions were conceptualized as extremely complex psychobiological systems which allow each individual to react flexibly and dynamically at both internal and external (environmental) contingencies [10]. A broader definition of emotions includes the somatic component and coordinated changes that are responses which involve the whole body at the level of physiology, behavior, or subjective experience [11]. The time and the evaluation process are also important emotional components that were considered in our study. The time can change both the evaluation results and the emotional process development (intensity, physiology). Emotions can be generated by the presence of a significant, immediate, real stimulus or by remembering this stimulus after a period of time, as well as by imagining an emotionally relevant situation [12].

In the last few years, a growing interest manifested in research on the emotional regulation mechanism involved in psychosomatic chronic diseases. In this context, there was evidence of the major presence of NA and ED in persons facing chronic diseases [13,14,15].

The ED process includes a large diversity of interrelated components, it therefore being difficult to find a unique and complete definition. The simplest and most accessible definition is formulated in opposition to the emotion regulation process and defines ED as “the inability to flexibly respond to and manage emotions” [16]. There are four processes mentioned in the literature that must be considered as the basis in defining ED: emotional awareness, emotional acceptance, impulse control, and the access to emotion regulation strategies when experiencing negative emotions [17,18]. When one or more of these components is blocked or difficult to use, we can talk about difficulties in emotion regulation or ED [19].

Vulnerability has been defined as “the degree to which a system, or part of it, may adversely react during the occurrence of a hazardous event”. This concept of vulnerability implies a measure of risk associated with the physical, social, and economic aspects, and the consequences derived from the system’s ability to cope with the resulting event [20]. A general explanation of vulnerability that captures and covers the three realms of human vulnerability could be the following: “vulnerability refers to a state of physical, emotional, and cognitive stability that is in danger of being disturbed or destroyed, due to being susceptible to destabilizing influences” [21].

The present work aims to assess the emotional mechanisms (ED, NA, and emotional vulnerability) involved in chronic diseases, applying a method inspired by Chaos Theory (Butterfly Effect) [22,23,24,25]. In Chaos Theory, the Butterfly Effect refers to a sensitive dependence on initial conditions, a very small change in the initial state being associated with large differences in later states [5,6,26]. The seemingly random system behaviors are the results of dynamics described by nonlinear differential or recurrence equations that could predict the system evolution in time [27,28,29,30,31]. In the emotional system, the Butterfly Effect can be applied as a low emotional stimulus in an initial state that can be associated with an important change in the system behavior over time [22]. The method, presented in one of our previous works [22], estimates the individual instability which occurs in the presence of a low disturbance, when the person evaluates his own emotional mechanisms. The method is based on monitoring the evolution in time of the distance between two identical systems with a small difference (perturbation) in the initial state [27,28,29,30,31]. The instability in evaluating one’s own emotional mechanisms in a past period of time was assessed by measuring and defining the “instability coefficient” Δ, the Euclidian distance between the vectors whose components are similar and reverted items of a test evaluating emotional mechanisms (Emotion Dysregulation Scale—DERS). Thus, Δ comprises the incongruence that occurs in the evaluation of one’s own ED mechanisms in the presence of an extremely weak stimulus and could be considered a “flavor” of the Lyapunov exponent [22,23].

This study analyzed the interaction between chronic disease (breast cancer, blood cancer, hypertension) and the main variables: ΔED, Δstrategies, Δawareness, Δgoals, Δimpulse, and Δclarity. ΔED represents a value of Δ estimated on all emotional dysregulation items as presented by DERS. The other Δ variables represent Δ values of ED components estimated on DERS clusters: strategies, awareness, goals, impulse, and clarity. Emotional vulnerability reflected by a fast growth (exponential) of the Δ values was associated with the chronic illness condition.

The main objective of the study was to assess if there is a significant relationship between the presence of the chronic disease and the “instability coefficients” Δ. The chronic disease group was compared with a healthy group in mean Δ values. One other important objective of the research was to investigate if the type of chronic disease is related to specific emotional mechanisms. For this purpose, the differences in Δ values among different chronic diseases were investigated. The third objective of the study was to analyze the differences in mean Δ values between each chronic disease group and a healthy group. A fourth objective was to investigate the prediction relationship between Δ and ED, as well as Δ and NA.

It was hypothesized that the mean Δ is higher in the chronic disease group than in the control group. Another important hypothesis was that mean Δ is significantly higher in illness groups with greater prognostic impact (cancer groups). The third hypothesis was that, depending on the type of chronic disease, there is a specific relation compared to the healthy persons: the patients with the low level of life prognostic (hypertension group) show similar values in mean Δ with the healthy persons. A fourth hypothesis was that there is a significant relationship between ΔED and ED, as well as between Δ and NA, among chronic disease groups. ΔED significantly predicts ED, while Δ significantly predicts NA.

## 2. Method

### 2.1. Sample

The sample consisted of 283 subjects, 189 females (67%), in a range of 19 to 77 years of age, mean age = 52.11 (SD = 12.099). The sample was divided into two research groups, matched as to age and gender. The clinical group (*n* = 137) of mean age = 53.30 (SD = 13.797) included subjects with psychosomatic chronic diseases (hypertension, blood cancer, breast cancer), while the control group (*n* = 146) of mean age = 51.06 (SD = 10.304) was made up of healthy subjects.

The study was conducted in “SS Annunziata” Clinical University Hospital, in the center of Italy, during the period January 2019–February 2020. The persons’ participation in the study was voluntary. Before the completion of the questionnaires, all participants signed the informed consent form. The patients came to the clinic to participate in the “Open Hospital Day” (weekly event providing greater information and sensibilization of chronic disease patients). They expressed their interest and completed the assessment protocol in the presence of a clinical psychologist experienced in emotion assessment. At the time of the evaluation, all patients involved in the study already had a diagnosis of chronic disease by a physician specialist. The questionnaire regarding the demographic data was completed at the end of the evaluation. The inclusion criteria were age (over 18) and education (>7 years). The exclusion criteria were the inability to complete the assessment for different reasons: age, culture, education (5 persons), or the failure to give informed consent (4 persons).

The breast cancer group, of mean age = 54.02 (SD = 10.327), included women having breast-conserving surgery as part of their treatment (98%), followed by hormonal medication (50%) concomitantly with immunotherapy (5%); chemotherapy (21%); no tumor medication (28%). The blood cancer group, of mean age = 53.86 (SD = 13.490), included patients diagnosed with lymphoma (62%), leukaemia (27%), myeloma (1%). The hypertension group, of mean age = 50.25 (SD = 18.036), was formed by patients diagnosed with high blood pressure.

The control group included persons without a diagnosis of chronic disease who expressed their interest in participating in the study. To analyze the differences between chronic disease groups and healthy people, the control group was divided into three subgroups matched as to number, age, and gender. The inclusion and exclusion criteria were the same as for the clinical group. No excluded research protocols were in the control group.

The demographic characteristics of the two groups (the clinical group and the healthy group) are presented in Table 1. The demographic and clinical characteristics of the illness groups are presented in Table 2.

### 2.2. Instruments

The research protocol consisted of self-reported questionnaires (validated in Italian population) assessing ED mechanisms, as well as the quality and type of emotions. [19,32,33,34]

Difficulties in Emotion Regulation Scale (DERS; Gratz and Roemer, 2004) [19,32] is a 36-item multidimensional self-report measure, subdivided into six subscales: 1. Nonacceptance of emotional responses (acceptance); 2. Goals—Difficulties engaging in goal-directed behavior, when experiencing negative emotions (goals); 3. Impulse—Impulse control difficulties, when experiencing negative emotions (impulse); 4. Awareness—Lack of emotional awareness (awareness); 5. Strategies—limited access to emotion regulation strategies that are perceived as effective (strategies); 6. Clarity—lack of emotional clarity (clarity), assessing the individual’s characteristic patterns of emotion dysregulation. Items are rated on a 5-point Likert-type scale (from 1 = almost never to 5 = almost always). The total score on DERS indicates a global value of ED. A high total score indicates a high level of ED. High scores indicate the presence of major difficulty in emotion regulation. In the current sample, internal consistency ranged between 0.74 for awareness and 0.82 for strategies and acceptance.

Reverse-scored items were numbered 1, 2, 6, 7, 8, 10, 17, 20, 22, 24, and 34. The measure yielded a total score (SUM), as well as scores on six subscales.

The scale evaluates the emotion dysregulation in clinical and healthy population by using the total score or different components of emotion dysregulation by using the scores on the six clusters [19,33].

Positive and Negative Affect Schedule of Trait (PANAS–TRAIT) is a version of the Positive and Negative Affect Schedule (PANAS; Watson, Clark, and Tellegen, 1988) that evaluates the emotions which one generally experiences (trait tendency to experience positive or negative emotions) [34]. Results were recorded as positive affect of trait (PAT) and negative affect of trait (NAT). Cronbach’s α in the present sample ranged between 0.70 for PAT and 0.86 for NAT.

Positive and Negative Affect Schedule of STATE (PANAS–STATE) is another version of the Positive and Negative Affect Schedule (PANAS; Watson, Clark, and Tellegen, 1988) that evaluates the emotions which one experiences in the assessment moment (positive or negative emotional reactivity in a specific situation) [34]. Results were recorded as positive affect of state (PAS) and negative affect of state (NAS). Cronbach’s α in the present sample ranged between 0.74 for PAS and 0.83 for NAS.

PANAS is a 20-item questionnaire: 10 items measure positive affect and 10 items measure negative affect. The positive affect: interested, excited, strong, enthusiastic, proud, alert, inspired, determinate, attentive, and active. The negative affect: distressed, upset, guilty, hostile, irritable, ashamed, nervous, jittery, and afraid. PANAS is one of the most widely used measures of affectivity, being reported to have excellent psychometric proprieties in Italian samples [22,23,35]. It was designed to measure affect in various contexts such as today, the past day, week, or year, or in general (on average). Thus, the scale can be used to measure state affect, dispositional or trait affect, emotional fluctuations throughout a specific period, or emotional responses to events. Each item is rated on a five-point Likert scale of 1 (not at all) to 5 (very much). High scores mean more frequent use of one kind of emotion. The measure has been used mainly as a research tool in group studies but can be applied within clinical and nonclinical populations as well. Using PANAS to evaluate emotions in two different temporal situations (e.g., in a past time and at present) allows the calculation of two global variables: NA (negative emotions) and PA (positive emotions). NA is obtained by summing NAT with NAS, and PA by summing PAT with PAS [34].

## 3. Procedure

The assessment of ED mechanisms in clinical and control groups was realized using a “flavor” of the Lyapunov function method. We employed the “instability coefficient” Δ, defined in our previous work [22]. Δ estimates the individual instability to a disturbance in evaluating their own emotional mechanisms. In this context, the dependence on initial conditions (Butterfly Effect) has been analyzed, starting from the idea that the effort of participating in the test is itself a small perturbation. This leads to inconsistent answers to equivalent questions and thus to incongruence in the evaluation of one’s own ED mechanisms [22]. Each similar item component also represents two different time points when the person self-evaluates her/his emotional mechanisms. The distance at the component level reflects the degree to which the person did not maintain a coherent response when asked to self-evaluate the emotional mechanisms in two different points in time:

(1) $$∆\;=\;\sqrt{\underset{i}{\sum }\;{\left(x_{i}-x_{ci}\right)}^{2}}\;\;\;\;\;$$

where *x_i_* is the item *i* score, and *x_ci_* is the score of the corresponding equivalent item or the inverted score of the corresponding reverted item [22].

The participants evaluated their difficulties in emotion regulation when answering the Difficulties in Emotion Regulation Scale (DERS) items. In order to minimize the side effects caused by the application conditions, the conditions provided in our previous work were followed [22]. The participants were informed about the evaluation aim, the typology of questionnaires, and about the order of completion. The information was provided by a clinical psychologist.

## 4. Statistical Analysis

All the variables (Δ, ED, NA. PAT, NAT, PAS, NAS) were investigated by descriptive analyses to determine means, standard deviations, and ranges. They were checked for normality in the chronic disease group (Table 3), in the healthy group (Table 4), and in the total sample (Table 5). The correlations between ED and Δ, NA and Δ, PAT and Δ, as well as NAT and Δ were assessed as primary analysis, using Pearson’s and Spearman’s coefficients. Six bivariate correlation analyses were calculated for each dependent variable (ED, NA, PAT, NAT). To avoid the type I error, all *p*-values were corrected for multi-testing, using the Bonferroni procedure (*p*-corrected value = 0.008) [36]. A series of *t*-tests and MANOVAs for dimensional variables were used to analyze the differences between groups. The *p*-values for the significance of *t*-tests were also corrected for multi-testing (Bonferroni *p*-corrected value = 0.008). The effect size Cohen’s d analysis was used to investigate how substantially different the variables were. The regression analysis was applied in order to verify both ED and NA prediction models.

## 5. Results

The relationship between age and the study variables (Δ, ED, NA, NAT, NAS, PAT, PAS), as well as between gender and the study variables, was assessed. The analysis of Pearson’s correlation coefficient and the independent samples *t*-test was performed. Age was not significantly correlated with the above-mentioned variables. No statistically significant differences were found between female and male subjects in the mean values of Δ, ED, NA, NAT, NAS, PAT, and PAS. The results are reported in the “Supplementary Materials” section (Tables S1 and S2).

### 5.1. Differences among Groups

The main objective of the study was to assess if there is a significant relationship between Δ and the presence of a chronic disease.

The first step was to investigate if significant differences were present in mean Δ values between the clinical group (chronic disease patients, *n* = 137) and the control group (healthy persons, *n* = 146). The independent sample *t*-test was performed to determine whether there was a statistically significant difference between the Δ means in these two groups. To investigate how substantially different the variables were, the Cohen’s d analysis was run. The results suggest that statistically significant differences in ΔED (t = 2.703, *p* = 0.007) and Δclarity (t = 4.293, *p* < 0.001) manifested between chronic disease patients and healthy persons. The mean Δ was significantly higher in the chronic disease group than in healthy persons. Further, Cohen’s effect size value suggests a moderate to high practical significance in Δclarity (d = 0.589), as well as a moderate practical significance in Δstrategies (d = 0.369) and ΔED (d = 0.399) (Table 6).

A multiple analysis of variance (one-way MANOVA) was performed in order to obtain further information about the statistical significance of the relationship between Δ and chronic disease. The presence of a chronic disease was considered as a categorical, independent variable and means of Δ as dependent variables. The results showed that there was a statistically significant difference in mean Δ values, based on the presence of the chronic disease: F(6, 229) = 3.912, *p* = 0.001, Pillai’s trace 0.103, partial η^2^ = 0.113. This result suggests that there is a significant difference across the levels of the independent variable (the presence of the chronic disease) on a linear combination of the dependent variables. The results showed that 11.3% of the variance in the dependent variables could be explained by the independent variable (Table 7).

One other important objective of our study was to investigate whether the type of chronic disease (breast cancer, blood cancer, and hypertension) influences Δ.

The multiple analysis of variance (one-way MANOVA) was performed to assess the main effects of chronic diseases on Δ. The type of chronic disease was considered as a categorical, independent variable, while the means of Δ (Δstrategies, Δawareness, Δimpulse, Δgoals, Δclarity, ΔED) were considered as multiple dependent variables. After the assumptions were verified, the results showed that there was a statistically significant difference in Δ, based on the type of the chronic disease: F(12, 206) = 2.977, *p* = 0.005, Pillai’s trace 0.284, partial η^2^ = 0.138. This result suggests that there is a significant difference across the levels of the independent variable (the type of chronic disease) on a linear combination of the dependent variables (mean Δ of different emotion dysregulation components and ΔED). The results showed that 13.8% of the variance in the dependent variables could be explained by the independent variable (Table 8).

The results of Schaffe post hoc tests, computed using alpha = 0.05, suggest that there is a statistically significant difference between the breast cancer group and the blood cancer group on the Δstrategies dependent variable (*p* = 0.008. Mean diff. = 1,137, SE = 0.363), while a moderate significant difference was found between the blood cancer group and the hypertension group on the Δimpulse dependent variable (*p* = 0.051, Mean diff. = 0.700, SE = 0.284). Moreover, a moderate statistically significant difference was reported between the breast cancer group and the hypertension group on the ΔED dependent variable (*p* = 0.018, Mean diff. = 1.303, SE = 0.451).

Applying the Bonferroni correction, only the differences in Δstrategies between the breast cancer group and the blood cancer group proved to have statistical significance.

The second step was to investigate if there were important differences in mean Δ between the illness groups (breast cancer, blood cancer, hypertension) and the healthy control subgroups. As specified in the sample section, the healthy group was divided into three control subgroups matched for gender, age, and number with clinical groups. The independent sample *t*-test and the effect size analysis Cohen’s d were performed to determine whether there was a statistically significant difference between the means of Δ in each pair of groups and how substantially different they were. The results showed that higher differences were reported between breast cancer patients and healthy persons (Table 9).

Applying the Bonferroni correction of *p*-value, three of six variables representing mean Δ values were significantly different between the two groups: ΔED (t = 4.913, *p* < 0.001), Δstrategies (t = 3.808, *p* < 0.001), and Δclarity (t = 5.559, *p* < 0.001). Thus, the mean Δ values were significantly higher in the breast cancer group than in healthy persons. This result suggests that women who were diagnosed with breast cancer reported greater difficulty than healthy persons in maintaining a coherent evaluation of their own emotional mechanisms. 

Further, Cohen’s effect size value suggests high practical significance in ΔED (d = 0.915) and Δclarity (d = 1.048), moderate to high significance in Δstrategies (d = 0.771), as well as moderate practical significance in Δimpulse (d = 0.436) and ΔED (d = 0.399). The practical significance in Δawareness (d = 0.212) was low (Table 9).

One of six variables was significantly different in mean Δ values between the blood cancer group and the control group: Δclarity (t = −3.423, *p* < 0.001). The mean Δclarity was significantly higher in the breast cancer group than in healthy persons. Cohen’s d effect size value suggests high practical significance in Δclarity (d = 0.760). This result suggests that the patients who were diagnosed with blood cancer reported greater difficulties than healthy persons in maintaining a coherent evaluation of their emotional clarity (Table 10).

Two of six variables—ΔED (t = −2.300, *p* < 0.05), Δimpulse (t = −2.225, *p* < 0.05)—showed moderate differences in mean Δ between the high blood pressure patients and the healthy persons. The Cohen’s d effect size value suggests moderate practical significance both in Δimpulse (d = 0.512) and in ΔED (d = 0.533). Applying the Bonferroni correction (*p* ≤ 0.008), the results did not satisfy the corrected *p*-value, showing no significant differences in mean Δ between hypertensive patients and healthy persons. The results of the independent sample *t*-test and Cohen’s d analysis are presented in Table 11.

### 5.2. Correlations between Variables

To investigate the relationship among Δ and the emotional components (ED, NA), the Pearson’s and Spearman’s bivariate correlations analyses were performed as the primary analysis. The correlations analyses were conducted between ED and the core variables of the study (ΔED, Δstrategies, Δawareness, Δimpulse, Δgoals, Δclarity). The correlations of NA, NAT, NAS with these variables were also analyzed. In interpreting the results, a Bonferroni corrected *p*-value (*p* ≤ 0.008) was considered.

In the chronic disease group, there was no statistically significant relationship between Δ and ED. Δ values showed no correlation with NA, as shown in Table S3. In the healthy group, ED was significantly correlated with ΔED (*r* = 0.379, *p* < 0.001) and with Δstrategies (*r* = 0.495, *p* < 0.001). There were significant correlations between NA and ΔED (*r* = −0.255, *p* = 0.004) and between NA and Δstrategies (*r* = 0.287, *p* = 0.001) (Table 12). 

In the breast cancer group, no significant correlations could be observed between the ED and the “instability coefficients” Δ. NA showed a negative significant correlation with Δclarity. Moreover, two variables which evaluate the type of emotion, PAT (*r* = −0.481, *p* = 0.001) and NAT (*r* = −0.454, *p* = 0.003), were significantly correlated with Δclarity, PAT being also significantly correlated with ΔED (r = −0.429, *p* = 0.005). These results suggest that high values of Δclarity are related to low values of NAT and to high values of PAT in breast cancer survivors, as shown in Table 13.

In the blood cancer group, ED was significantly correlated with ΔED (*r* = −0.426, *p* = 0.008), while NA was significantly correlated with ΔED (*r* = −0.516, *p* = 0.001), Δimpulse (*r* = −0.454, *p* = 0.005), Δgoals (*r* = −0.436, *p* = 0.007), and Δclarity (*r* = −0.435, *p* = 0.007) (Table 14). These results showed that high values of ED were associated with low values of ΔED in the blood cancer group, while high values of NA were associated with low values of the “instability coefficients” (Δimpulse, Δgoals, and Δclarity). No significant correlations could be seen between ED and Δ or between NA and Δ in the hypertension group, as shown in Table S4.

### 5.3. Regression Models

To investigate the relationship between ED and ΔED, a multiple linear regression analysis was performed, heaving ED as dependent variable and ΔED, NAT, NAS as independent variables. The results of multiple regression suggest that the model better explains the ED variance in the blood cancer group (24%), followed by the hypertension group (46%). In the breast cancer group, the model was not significant. A summary of multiple standard regression results is presented in Table 15. 

As unique independent variable, ΔED explains 18% from the ED variance (F (1, 48) = 9.885, *p* = 0.008, R^2^ = 0.18) in the blood cancer group. 

To verify if there is an important relationship between the “instability coefficients” Δ and NA, another multiple linear regression analysis has been run, having NA as dependent variable and Δclarity, Δimpulse, Δstrategies as independent variables. The results suggest that the relationship between Δ and NA may be important. This model significantly predicts NA in all the three illness groups. The model better explains the NA variance in the blood cancer group (29%), followed by the breast cancer group (17%), and the hypertension group (11%). A synthesis of the multiple regression results is presented in Table 16.

## 6. Discussions

Emotion regulation represents the individual’s capacity to experience, process, and modulate emotional response [12]. Chronic disease represents a new physical, psychological, and medical condition to which the patient must adapt, requiring significant emotional resources [15]. When the adaptive emotional mechanisms are missing, the everyday self-care management of chronic disease overwhelms the emotional demands, depleting an individual’s resources. Deficits or malfunctioning of emotion regulation mechanisms lead to emotion dysregulation—ED—and high levels of negative affect—NA—that are shown to have a serious impact on behaviors, wellbeing, and an individual’s quality of life, determining poor health outcomes [12,37].

In the present study, we realized an assessment of emotional mechanisms (ED, NA, emotional vulnerability) in psychosomatic diseases, starting from the conceptualizations of emotions as complex dynamic systems that could be investigated, understood, and predicted using concepts and proprieties from the Complex Systems Theory (Chaos Theory) [9,10]. We tried to connect these three emotional mechanisms in different groups of psychosomatic diseases (blood cancer, breast cancer, hypertension), as well as in a group of healthy people, with high dependence on initial conditions (Butterfly Effect), specific to a large class of nonlinear dynamical systems [24,25]. We calculated the “instability coefficient” Δ, defined in one of our previous works as the Euclidian distance between the pairs of vectors whose components are similar or reverted items of a test measuring ED. Δ comprises the incongruence that occurs in the evaluation of one’s own ED mechanisms in the presence of an extremely weak stimulus and could be considered a “flavor” of the Lyapunov exponent [22,23].

The main goal of the study was to analyze the differences in mean Δ values between the chronic disease group and the healthy group, as well as among the chronic illness groups. The results suggest that highly significant differences in Δclarity and ΔED were found between the clinical group (chronic diseases) and the control group (healthy persons). People facing a chronic illness showed major difficulties in maintaining a coherent evaluation of their emotional clarity and in their efficiency to regulate negative emotions.

The differences in Δ values among the three disease groups, as well as between illness groups and healthy persons, were analyzed. The results confirmed our hypothesis: higher values of Δ corresponded to the chronic disease group, being higher in illness groups with greater prognostic impact (cancer groups). This relationship could suggest a major emotional vulnerability in these patients and may be related to certain illness characteristics: life prognosis, the impact on personal life, and subjective wellbeing.

Highly significant differences resulted also between breast cancer women and healthy women in Δstrategies, Δclarity, and ΔED, meaning that the former group of patients reported major difficulties in maintaining an accurate evaluation of their capacity of applying appropriate regulation strategies compared to healthy women. Moreover, the mean Δclarity values were radically different between the blood cancer group and the healthy group, suggesting that persons experiencing a blood cancer disease reported more difficulties in evaluating accurately their emotional mechanisms, when living negative emotions.

No highly significant differences were found in Δ values between hypertensive patients and healthy people. This result proves that the hypertensive patients and the healthy people manifested almost similar difficulties in maintaining an accurate evaluation of their own emotional mechanisms.

One other goal of the study was to investigate the relationship between Δ and two important emotional components, ED and NA, which are related to chronic disease. The results suggest that the 18% of ED variance in persons diagnosed with blood cancer could be explained by their difficulties in constantly maintaining a coherent evaluation of their own emotional mechanisms. Meanwhile, in other disease groups (breast cancer, hypertension), this relation was not statistically significant.

An interesting result was obtained by analyzing the prediction relationship between Δclarity, Δimpulse, and Δstrategies as independent variables and NA as a dependent variable. It was proven that the model significantly explained the NA variance in the blood cancer group (30%), the breast cancer group (17%), and the hypertension group (11%). The patients who manifested difficulties in accurately evaluating their emotions, their control of impulses, and their efficiency of emotional management strategies may experience higher levels of NA than other people when facing a chronic disease.

In the last few years, the physics theories of Chaos and Complexity proved to be important elements in the complex changes in health and care models [22,23,24,25]. For this reason, we propose an interdisciplinary chaos analysis of ED, NA, and emotional vulnerability in psychosomatic chronic diseases, hypothesizing a significant relationship between emotional vulnerability and psychosomatic disease. We started from the conceptualization of emotions as a complex dynamic system that can be investigated and understood using mathematics laws (nonlinear coupled differential equations). The emotional system and somatic system were considered as two complex dynamical systems in interaction [5,38]. Due to this interaction and to the complexity laws, a small perturbation in an inner state of the emotional system could generate an important (exponential) reaction in the somatic system over time. The emotional vulnerability was conceptualized as the risk that a system could fail to cope with a hazardous event, adversely reacting during the occurrence of this event. The vulnerability was analyzed using the “instability coefficients” Δ values: high Δ values reflect a major emotional vulnerability of a stimulus, being associated with the chronic disease condition [22].

Even though the results of the present study are promising in proposing a method useful in a new type of exploration and understanding emotional mechanisms, the limits of our research need to be taken into consideration. The application of complex system dynamics theory in the prediction and understanding of emotional functioning is in its early stage. Consequently, these results need caution in interpretation, not being enough to give a diagnosis only by themselves. They can rather offer detailed knowledge on emotional mechanisms, not accessible in the classical approach. Future research could fruitfully explore these issues in different groups of the population and assess a higher variety of emotional mechanisms, offering specialists better competences to guide patients in their emotional disease management.

## 7. Conclusions

As a conclusion of this entire research, significant differences in Δ values have been shown among different groups of chronic diseases, as well as compared to those of the healthy group. Thus, breast cancer patients reported higher Δ values compared to the other illness groups, as well as compared to healthy persons. Another achievement of this study is that Δ values have been found to significantly predict ED and NA, two important emotional mechanisms in chronic diseases.

Deficits or malfunctioning of emotion regulation mechanisms lead to ED and raise the levels of NA, proven to highly impact behaviors, wellbeing, and the individuals’ life quality, causing poor health outcomes. These mechanisms are involved in chronic disease onset, course, and treatment, helping health professionals to better understand and manage long-term interventions.

Compared to the classical approaches, the original contribution of our research is that these results encourage us to propose this interdisciplinary method of assessment as a challenging, valid tool of investigation, understanding, and prediction of complex phenomena that occur in the emotional and somatic system.

## Figures and Tables

**Table 1:** Demographic characteristics of the clinical group.

Groups	*n*	Age (Years)	Age (Years)	Age (Years)	Gender	Working Status	Civil Status	Education (Years)
		Min	Max	Mean ± SD	Female *n* (%)	Working *n* (%)	Married *n* (%)	Mean ± SE
Chronic disease	137	19	77	53.30 ± 13.797	95 (68.5)	91 (64.24)	101 (76.21)	11.43 ± 3.92
Healthy persons	146	22	77	51.06 ± 10.304	92 (65.6)	101 (76.14)	106 (82.34)	10.93 ± 2.92

**Table 2:** Patients’ demographic characteristics and illness characteristics in illnesses groups.

Demographic Characteristics	Breast Cancer	Blood Cancer	Hypertension
	***n* = 50**	***n* = 46**	***n* = 41**
Age (Years) Mean ± SD	54.02 ± 10.327	53.86 ± 13.490	50.25 ± 18.036
Gender Female (%)	50 (100%)	30 (62%)	26 (62%)
Civil status Married *n* (%)	37 (73%)	38 (82%)	29 (71%)
Education Years (Mean)	10.02	11.87	10.95
Illness Time Years (Mean)	3.5	1.86	6.56
Illness (Characteristics)	99% Surgical breast intervention	62% Lymphoma 27% Leukemia 1% Myeloma	100% High blood pressure
Medication	28% No tumoral medication 21% Chemotherapy 50% Hormonal 5% Immunotherapy (concomitant)	100% Chemotherapy+ 44% autologous bone marrow transplant (concomitant)	100% Antihypertensive medication

**Table 3:** Descriptive statistics/chronic disease group (*n* = 137).

Variables	Min	Max	Mean	SD	Skewness	Std. Error	Kurtosis	Std. Error
ΔED	2.236	15.524	5.172	1.985	1.557	0.229	5.450	0.455
Δstrategies	0.000	14.142	2.440	1.678	2.210	0.229	12.428	0.455
Δawareness	0.000	5.656	1.542	1.128	0.743	0.229	0.858	0.455
Δimpulse	0.000	5.000	2.084	1.204	0.273	0.229	−0.606	0.455
Δgoals	0.000	5.000	1.716	1.314	0.558	0.229	−0.501	0.455
Δclarity	0.000	5.656	2.433	1.386	0.405	0.229	−0.492	0.455
ED	43	121	81.25	16.718	0.179	0.229	−0.126	0.455
NA	17	94	40.66	14.035	0.686	0.229	1.044	0.455
PAT	13	44	30.37	6.066	−0.095	0.229	−0.247	0.455
NAT	10	47	19.71	6.860	1.172	0.229	1.961	0.455
PAS	13	39	27.56	5.646	−0.253	0.229	−0.218	0.455
NAS	10	47	21.53	8.724	0.526	0.229	0.456	0.455

ΔED—a value of Δ estimated on all emotional dysregulation items as presented by DERS; Δstrategies—a value of Δ estimated on the items of the cluster strategies; Δawareness—a value of Δ estimated on the items of the cluster awareness; Δimpulse—a value of Δ estimated on the items of the cluster impulse; Δgoals—a value of Δ estimated on the items of the cluster goals; Δclarity—a value of Δ estimated on the items of the cluster clarity; ED—emotion dysregulation; NA—negative affect; PAT—positive affect of trait; NAT—negative affect of trait; PAS—positive affect of state; NAS—negative affect of state.

**Table 4:** Descriptive statistics/healthy group (*n* = 146).

Variables	Min	Max	Mean	SD	Skewness	Std. Error	Kurtosis	Std. Error
ΔED	1.414	9.055	4.544	1.572	0.386	0.217	−0.913	0.430
Δstrategies	0.000	5.744	1.941	1.361	0.732	0.217	−0.051	0.430
Δawareness	0.000	4.472	1.436	1.136	0.556	0.217	−0.254	0.430
Δimpulse	0.000	5.656	1.967	1.244	0.596	0.217	0.189	0.430
Δgoals	0.000	4.472	1.656	1.257	0.594	0.217	−0.424	0.430
Δclarity	0.000	5.656	1.696	1.251	0.768	0.217	0.446	0.430
ED	36	121	67.22	17.674	0.852	0.117	0.513	0.430
NA	20	67	32.86	11.140	1.050	0.217	0.592	0.430
PAT	11	50	31.62	7.392	−0.300	0.217	0.590	0.430
NAT	10	36	17.65	6.577	0.986	0.217	0.446	0.430
PAS	11	50	29.34	7.696	−0.077	0.217	−0.158	0.430
NAS	10	34	15.21	5.660	1.277	0.217	1.083	0.430

**Table 5:** Descriptive statistics/total sample (*n* = 283).

Variables	Min	Max	Mean	SD	Skewness	Std. Error	Kurtosis	Std. Error
ΔED	1.414	15.524	4.839	1.802	1.219	0.158	4.359	0.316
Δstrategies	0.000	14.142	2.176	1.535	2.310	0.158	14.541	0.316
Δawareness	0.000	5.656	1.486	1.131	0.636	0.158	0.243	0.316
Δimpulse	0.000	5.656	2.022	1.224	0.443	0.158	−0.211	0.316
Δgoals	0.000	5.000	1.684	1.282	0.575	0.158	−0.472	0.316
Δclarity	0.000	5.656	2.043	1.364	0.587	0.158	−0.165	0.316
ED	36	121	73.82	18.571	0.397	0.158	−0.329	0.316
NA	17	94	36.53	13.150	0.911	0.158	0.999	0.316
PAT	11	50	31.03	6.815	−0.179	0.158	0.378	0.316
NAT	10	47	18.62	6.777	1.055	0.158	1.219	0.316
PAS	11	50	28.52	6.866	−0.010	0.158	0.318	0.316
NAS	10	47	18.14	7.886	1.010	0.158	0.392	0.316

**Table 6:** Differences between groups: independent samples *t*-test and Cohen’s d coefficient. Gr. 1: chronic disease, *n* = 137; Gr. 2: healthy persons, *n* = 146.

Variables	Chronic Diseases		Healthy				
	Mean	SD	Mean	SD	*p*	t	Cohen’s d
Δstrategies	2.440	1.678	1.941	1.361	0.013	2.516	0.369
Δawareness	1.542	1.128	1.436	1.136	0.474	0.717	0.093
Δimpulse	2.084	1.204	1.967	1.244	0.464	0.734	0.096
Δgoals	1.716	1.314	1.656	1.257	0.722	0.356	0.046
Δclarity	2.433	1.386	1.696	1.251	**0.000**	**4.293**	**0.589**
ΔED	5.172	1.985	4.544	1.572	**0.007**	**2.706**	**0.399**

Differences are significant at the 0.008 level (Bonferroni adjusted *p*-value). The values that are significant after correction for multi-testing are indicated in the table in boldface.

**Table 7:** Differences between groups (one-way MANOVA); tests of between-subjects effects. Gr. 1: chronic disease, *n* = 137; Gr. 2: healthy persons, *n* = 146.

Variables	Mean Square	F	Sig.	Partial Eta Square	Observed Power *
Δstrategies	14.606	6.332	0.013	0.047	0.707
Δawareness	0.660	0.514	0.474	0.004	0.110
Δimpulse	0.806	0.537	0.464	0.004	0.113
Δgoals	0.210	0.127	0.722	0.003	0.065
Δclarity	31.942	**18.432**	**0.000**	**0.098**	**0.990**
ΔED	23.157	**7.320**	**0.007**	**0.067**	**0.769**

* computed using alpha = 0.05. The values that are significant after correction for multi-testing are indicated in the table in boldface.

**Table 8:** Differences between groups (one-way MANOVA); tests of between-subjects effects.

Variables	Breast Cancer	Blood Cancer	Hypertension					
	Mean	Mean	Mean	Mean Square	F	Sig.	Partial Eta Square	Observed Power *
Δstrategies	3.080	1.943	2.174	14.300	**5.487**	**0.005**	**0.122**	**0.841**
Δawareness	1.707	1.501	1.371	1.071	0.839	0.435	0.035	0.191
Δimpulse	2.227	2.322	1.622	4.897	3.532	0.033	0.081	0.647
Δgoals	2.012	1.670	1.380	3.681	2.176	0.118	0.059	0.437
Δclarity	2.707	2.499	1.999	4.672	2.497	0.087	0.044	0.492
ΔED	5.862	4.916	4.560	17.229	4.660	0.011	0.119	0.774

* computed using alpha = 0.05. The values that are significant after correction for multi-testing are indicated in the table in boldface.

**Table 9:** Differences between groups: independent samples *t*-test and Cohen’s d coefficient. Gr.1: breast cancer patients, *n* = 50; Gr.2: healthy persons, *n* = 53.

Variables	Breast Cancer		Healthy				
	Mean	SD	Mean	SD	*p*	t	Cohen’s d
Δstrategies	3.080	2.211	1.668	1.051	**0.000**	**3.808**	**0.771**
Δawareness	1.707	1.098	1.555	1.143	0.025	2.282	0.212
Δimpulse	2.227	1.205	1.757	1.171	0.070	1.833	0.436
Δgoals	2.012	1.315	1.424	1.138	0.029	2.219	0.344
Δclarity	2.707	1.354	1.365	0.835	**0.000**	**5.559**	**1.048**
ΔED	5.862	2.311	3.871	1.341	**0.000**	**4.913**	**0.915**

Differences are significant at the 0.008 level (Bonferroni adjusted *p*-value). The values that are significant after correction for multi-testing are indicated in the table in boldface.

**Table 10:** Differences between groups: independent samples *t*-test and Cohen’s d coefficient. Gr. 1: blood cancer patients, *n* = 46; Gr. 2: healthy persons, *n* = 49.

Variables	Blood Cancer		Healthy				
	Mean	SD	Mean	SD	*p*	t	Cohen’s d
Δstrategies	1.943	0.961	1.785	1.255	0.524	−0.641	0.143
Δawareness	1.501	1.185	1.639	1.178	0.602	0.524	0.116
Δimpulse	2.322	1.229	1.909	1.316	−1.457	0.149	0.324
Δgoals	1.670	1.287	1.810	1.362	0.638	0.472	0.105
Δclarity	2.499	1.361	1.519	1.213	**0.001**	**−3.423**	**0.760**
ΔED	4.916	1.882	4.530	1.577	0.318	−1.006	0.222

Differences are significant at the 0.008 level (Bonferroni adjusted *p*-value). The values that are significant after correction for multi-testing are indicated in the table in boldface.

**Table 11:** Differences between groups: independent sample *t*-test and Cohen’s d coefficient. Gr.1: hypertensive patients, *n* = 41, Gr.2: healthy persons, *n* = 44.

Variables	Hypertension		Healthy				
	Mean	SD	Mean	SD	*p*	t	Cohen’s d
Δstrategies	2.174	1.239	2.406	1.596	0.482	−0.707	0.162
Δawareness	1.371	1.104	1.520	1.075	0.559	−0.587	0.136
Δimpulse	1.622	1.074	2.229	1.284	0.029	−2.225	0.512
Δgoals	1.380	1.295	1.820	1.295	0.150	−1.456	0.339
Δclarity	1.999	1.392	2.125	1.493	0.707	−0.377	0.087
ΔED	4.560	1.302	5.288	1.425	0.024	−2.300	0.533

Differences are significant at the 0.008 level (Bonferroni adjusted *p*-value).

**Table 12:** Correlations between variables (Pearson’s coefficients): Δ/ED; Δ/NA; healthy group (*n* = 146); (Δ—“instability coefficients”; ED—emotion dysregulation; NA—negative affect).

Variables	ED		NA	
	*r*	*p*	*r*	*p*
**ΔED**	0.379 **	**0.000**	0.255 **	**0.004**
**Δstrategies**	0.495 **	**0.000**	0.287 **	**0.001**
**Δawareness**	0.048	0.594	0.202	0.024
**Δimpulse**	0.216	0.016	0.093	0.301
**Δgoals**	0.093	0.304	0.011	0.903
**Δclarity**	0.208	0.020	0.102	0.258

** Correlation is significant at the 0.008 level (Bonferroni adjusted *p*-value). The values that are significant after correction for multi-testing are indicated in the table in boldface.

**Table 13:** Correlations between variables (Pearson’s coefficients): Δ/ED; Δ/NA; breast cancer group (*n* = 50); Δ—“instability coefficients”; ED—emotion dysregulation; NA—negative affect.

Variables	ED		NA		PAT		NAT	
	*r*	*p*	*r*	*p*	*r*	*p*	*r*	*p*
**ΔED**	−0.176	0.264	−0.282	0.070	0.429 **	**0.005**	−0.265	0.090
**Δstrategies**	−0.077	0.626	−0.147	0.352	0.220	0.162	−0.115	0.469
**Δawareness**	−0.134	0.396	−0.168	0.288	0.075	0.636	−0.172	0.277
**Δimpulse**	−0.100	0.530	−0.219	0.164	0.331	0.032	−0.256	0.102
**Δgoals**	−0.001	0.897	0.153	0.334	0.113	0.476	−0.169	0.285
**Δclarity**	−0.253	0.106	−0.440 **	**0.004**	0.481 **	**0.001**	−0.454 **	**0.003**

** Correlation is significant at the 0.008 level (Bonferroni adjusted *p*-value). The values that are significant after correction for multi-testing are indicated in the table in boldface.

**Table 14:** Correlations between variables (Pearson’s coefficients): Δ/ED; Δ/NA; blood cancer group (*n* = 46); (Δ—“instability coefficients”; ED—emotion dysregulation; NA—negative affect).

Variables	ED		NA	
	*r*	*p*	*r*	*p*
**ΔED**	0.426 **	**0.008**	–0.516 **	0.001
**Δstrategies**	–0.225	0.181	–0.212	0.207
**Δawareness**	–0.190	0.261	–0.076	0.654
**Δimpulse**	–0.168	0.321	–0.454 **	**0.005**
**Δgoals**	–0.351	0.033	–0.436 **	**0.007**
**Δclarity**	–0.393	0.016	–0.435 **	**0.007**

** Correlation is significant at the 0.008 level (Bonferroni adjusted *p*-value). The values that are significant after correction for multi-testing are indicated in the table in boldface.

**Table 15:** Standard regression analysis summary for variables predicting Emotion Dysregulation (ED) in blood cancer and hypertension groups.

Group	Model	R^2^	F	*p*	Β (ΔED)
Blood cancer	ΔED, NAT, NAS	0.241	3.137	0.016 *	–0.201
Hypertension	ΔED, NAT, NAS	0.462	8.019	0.001 **	0.095

* *p* < 0.05; ** *p* < 0.001.

**Table 16:** Standard regression analysis summary for variables predicting Emotion Dysregulation (ED) in different groups.

Group	R^2^	B	SEB	*p*
Blood cancer	0.292	–3.403	1.346	0.016 *
Breast cancer	0.171	5.93	2.531	0.003 *
Hypertension	0.112	6.582	2.250	0.006 *

* *p* < 0.05. SEB—Standard error of B.

## Supplementary Materials

The following are available online at https://www.mdpi.com/2076-3425/10/10/673/s1, Table S1: Correlations between variables (Pearson’s and Spearman’s coefficients), Table S2: Differences between groups (Independent Samples T-Test), Table S3: Correlations between variables (Pearson’s and Spearman’s coefficients), Table S4: Correlations between variables (Pearson’s coefficients).
