# Supplementary material for: Emotional Dysregulation Mechanisms in Psychosomatic Chronic Diseases Revealed by the Instability Coefficient
Source: Brain Sci. 2020 Sep 25;10(10):673. doi: 10.3390/brainsci10100673 (PMC7601642; doi:10.3390/brainsci10100673)
Supplement: Supplementary file 1 [file brainsci-10-00673-s001.pdf]

brain sciences

Supplementary materials

Table S1. Correlations between variables (Pearson's and Spearman's coefficients); AGE/Δ/ED/NA/PAT/NAT/PAS/NAS; (Δ—"Instability Coefficients", ED—Emotion Dysregulation, NA—Negative Affect, PAT—Positive Affect of Trait, NAT – Negative Affect of Trait, PAS — Positive Affect of State, PAT—Positive Affect of State).

	AGE		AGE	
	**Mean**	**SD**	***r***	***p***
**ΔED**	4.839	1.802	0.031	0.639
**Δstrategies**	2.176	1.535	0.049	0.450
**Δawareness**	1.486	1.131	0.005	0.934
**Δimpulse**	2.022	1.224	0.030	0.650
**Δgoals**	1.684	1.282	−0.042	0.517
**Δclarity**	2.043	1.364	0.111	0.089
**ED**	73.823	18.571	0.111	0.090
**NA**	36.53	13.15	−0.068	0.301
**PAT**	31.03	6.815	−0.090	0.166
**NAT**	18.62	6.777	− 0.109	0.093
**PAS**	28.52	6.866	0.072	0.272
**NAS**	18.14	7.886	−0.002	0.976

Table S2. Differences between groups (Independent Samples T-Test). Gender groups: Gr. 1 - Females; Gr. 2 – Males.

	Females N = 189		Males N = 94			
	**Mean**	**SD**	**Mean**	**SD**	**t**	***p***
ΔED	4.471	1.832	5.039	1.733	−1.196	0.233
Δstrategies	2.235	1.638	2.056	1.304	0.837	0.403
Δawareness	1.439	1.074	1.579	1.240	−0.849	0.374
Δimpulse	1.967	1.216	2.135	1.239	−0.986	0.322
Δgoals	1.679	1.244	1.694	1.362	−0.084	0.931
Δclarity	1.867	1.323	2.199	1.385	−2.815	0.009
ED	74.19	19.574	73.08	16.448	0.432	0.647
NA	36.70	13.584	36.18	12.300	0.283	0.770
PAT	31.25	6.086	30.59	8.120	0.632	0.487
NAT	18.85	6.805	18.15	6.038	0.740	0.458
PAS	29.33	6.250	26.83	7.766	2.451	0.016
NAS	17.96	7.870	18.50	7.961	−0.486	0.626

**. The difference is significant at the $p \leq 0.004$ level (Bonferroni adjusted p-value).

Table S3. Correlations between variables (Pearson's and Spearman's coefficients); Δ/ED; Δ /NA. Chronic Disease Group (N = 137); (Δ = "Instability Coefficients"; ED = Emotion Dysregulation; NA = Negative Affect).

	ED		NA	
	r	***p***	***r***	***p***
**ΔED**	−0.027	0.777	−0.206	0.030
**Δstrategies**	0.081	0.401	−0.100	0.298
**Δawareness**	−0.058	0.544	−0.050	0.599

**Δimpulse**	0.075	0.435	−0.163	0.087
**Δgoals**	0.011	0.812	−0.071	0.460
**Δclarity**	−0.017	0.860	−0.152	0.112

** Correlation is significant at the 0.008 level (Bonferroni adjusted *p*-value).

Table S4. Correlations between variables (Pearson's coefficients); Δ/ED; Δ/NA; .Hypertension Group (N = 41); (Δ—"Instability Coefficients"; ED—Emotion Dysregulation; NA = Negative Affect).

	**ED**		**NA**	
	r	***p***	***r***	***p***
**ΔED**	0.090	0.625	0.237	0.192
**Δstrategies**	0.113	0.538	0.109	0.553
**Δawareness**	−0.071	0.700	0.096	0.602
**Δimpulse**	0.208	0.254	−0.001	0.894
**Δgoals**	0.013	0.942	−0.172	0.346
**Δclarity**	0.207	0.257	0.275	0.127

** Correlation is significant at the 0.008 level (Bonferroni adjusted *p*-value).
